# Technical Innovations and Complex Cases in Robotic Surgery for Lung Cancer: A Narrative Review

**DOI:** 10.3390/curroncol32050244

**Published:** 2025-04-22

**Authors:** Giacomo Cusumano, Giuseppe Calabrese, Filippo Tommaso Gallina, Francesco Facciolo, Pierluigi Novellis, Giulia Veronesi, Stefano Viscardi, Filippo Lococo, Elisa Meacci, Alberto Terminella, Gaetano Romano, Cristina Zirafa, Franca Melfi, Stefano Margaritora, Marco Chiappetta

**Affiliations:** 1 General Thoracic Surgery Unit, Azienda Ospedaliero Universitaria Policlinico “G. Rodolico-San Marco”, 95100 Catania, Italy; giacomo.cusumano@unict.it (G.C.); albertoterminella0@gmail.com (A.T.); 2 Department of Surgery and Medical-Surgical Specialties, University of Catania, 95100 Catania, Italy; 3 Thoracic Surgery, Fondazione Policlinico Universitario A. Gemelli IRCCS, 00168 Rome, Italy; filippo.lococo@policlinicogemelli.it (F.L.); elisa.meacci@policlinicogemelli.it (E.M.); stefano.margaritora@policlinicogemelli.it (S.M.); 4 Department of General Thoracic Surgery, Fondazione Policlinico Universitario “A. Gemelli”, IRCCS, Università Cattolica del Sacro Cuore, 00168 Rome, Italy; 5 Department of Thoracic Surgery, IRCCS Regina Elena National Cancer Institute, 00144 Rome, Italy; filippogallina92@gmail.com (F.T.G.); francesco.facciolo@ifo.gov (F.F.); 6 School of Medicine, Vita-Salute San Raffaele University, 20123 Milano, Italy; pierluigi.novellis84@gmail.com (P.N.); veronesi.giulia@hsr.it (G.V.); viscardi.stefano@hsr.it (S.V.); 7 Department of Thoracic Surgery, IRCCS San Raffaele Scientific Institute, 20123 Milan, Italy; 8 Minimally Invasive and Robotic Thoracic Surgery, University Hospital of Pisa, 56124 Pisa, Italy; gaetano_romano1986@hotmail.com (G.R.); c.zirafa@gmail.com (C.Z.); 9 Department of Pharmacy, Health and Nutrition Sciences, University of Calabria (UniCal), 87036 Rende, Italy; franca.melfi@unipi.it; 10 Surgical Robotic Center, University Hospital of Pisa, 56124 Pisa, Italy; 11 Thoracic Surgery, Magna Graecia University, 88100 Catanzaro, Italy; marcokiaps@hotmail.it

**Keywords:** non-small cell lung cancer, robotic surgery, RATS, mini-invasive surgery

## Abstract

For over two decades, robotic-assisted thoracic surgery (RATS) has revolutionized thoracic oncology. With enhanced visualization, dexterity, and precision, RATS has reduced blood loss, shortened hospital stays, and sped up recovery compared to traditional surgery or video-assisted thoracoscopic surgery (VATS). The use of 3D high-definition imaging and articulated instruments allows for complex resections and advanced lymph node assessment. RATS delivers oncological outcomes similar to open surgery and VATS, with high rates of complete (R0) resections and acceptable complication rates. Its minimally invasive nature promotes quicker recovery. Advances in imaging software and augmented reality further enhance surgical accuracy and reduce intraoperative risks. However, RATS has some limitations, including high costs and a lack of tactile feedback, and certain complex procedures, such as extended resections and intrapericardial interventions, remain challenging. With growing experience and technological advances, RATS shows promise in reducing morbidity, improving quality of life, and expanding access to advanced oncologic care. This article reviews the evolution, benefits, and limitations of RATS in NSCLC treatment, highlighting its emerging role in managing complex cases.

## 1. Introduction

Over two decades after the first robotic lung lobectomy for lung cancer, numerous studies have highlighted the benefits and advantages of integrating robotics into thoracic surgery (RATS) [1]. The robotic technique is undeniably recognized for its improved and magnified visualization of anatomical structures, thanks to high-definition 3D imaging and realistic depth perception. Additionally, the use of articulated instruments with wide ranges of motion, combined with the elimination of intentional tremors, ensures greater dexterity compared to video-assisted thoracoscopic surgery (VATS), allowing for the performance of complex maneuvers in confined spaces.

The clinical advantages, including faster functional recovery, reduced blood loss, shorter hospital stays, and a less steep learning curve for surgeons compared to laparoscopic techniques, have contributed to the widespread adoption of this technology [2]. While robotics was initially more prevalent in urology and other fields, its early adoption facilitated the availability of robots for thoracic surgery. Today, robotic surgery in thoracic procedures is a standard of care, particularly for mediastinal diseases and pulmonary surgery. To date, various lung RATS resections for lung cancer have been realized, and the experience ranges from segmental resections to pneumonectomies and robotic lobectomies.

In the more than 20 years since the first cases of robotic lobectomies for lung cancer, there has been significant refinement of robotic techniques and technological advancements, with the introduction of more advanced robotic models. The oncological outcomes are comparable to those of open lobectomy and are similar to VATS lobectomy in terms of oncological efficacy and functional benefits [3].

Furthermore, recent experiences indicate that the robotic approach led to improved lymph node assessment [4]. The new well-established uniportal thoracoscopic approach has opened the possibility of performing major lung resections through a single incision. Similarly, early experiences with biportal and uniportal robotic pulmonary resections have been described, enabled by new robotic technologies [5,6]. Among all, the work published by Kuzmych and colleagues [7], who described in 2024 the approach in biportal RATS with the use of green indocyanine to perform robotic anatomical segmentectomies, seems very promising.

Despite these advantages, the real-world application of robotics in major lung resections has been limited. The main debates focus on longer operative times, the direct and indirect costs of robotic surgery compared to open and VATS approaches, and certain technical challenges, such as the lack of tactile feedback and the feasibility of performing all types of procedures, including extended resections and surgeries following induction therapy [2].

This narrative review explores the main approaches and outcomes of robotic-assisted lobectomy (RATS) for lung cancer, with a particular focus on the potential benefits and challenges of complex resections and post-induction surgery.

It also outlines the state-of-the-art in RATS resections for lung cancer, highlighting innovative experiences with new biportal and uniportal RATS approaches and the new potential applications of preoperative and intraoperative applications of software in robotic surgery.

## 2. State of the Art

The earlier experiences of robotic applications in thoracic surgery marked a transformative phase in the evolution of minimally invasive surgery. Starting in the early 2000s, robotic systems—most notably the da Vinci Surgical System—were first adapted for use in thoracic procedures following their success in urological and gynecological surgeries. The pioneering experiences in thoracic surgery, particularly in the field of thymic resection and major lung resections, have been significantly influenced by the work of Melfi and collaborators [8]. Their early work, published in 2002, involved a variety of procedures, including thymic resections and lobectomies, demonstrating the feasibility and safety of robotic approaches in complex thoracic cases. Their contributions have advanced minimally invasive approaches and robotic surgery, pushing the boundaries of what is achievable in these complex procedures. A significant challenge was adapting the robotic system to the thoracic anatomy while developing a reproducible, ergonomic, and safe surgical technique. Moreover, it was essential to anticipate a learning curve to gain proficiency in operating the robotic system within the chest and handling delicate structures like the lung parenchyma and pulmonary vessels, despite the lack of tactile feedback. Melfi and colleagues published in 2008 details the application of robotic surgery in lobectomy for lung cancer, presenting outcomes and the learning curve associated with the refined technique [9]. Other centers in Italy are making significant strides in the application of robotics in thoracic surgery. In Milan, the research conducted by Veronesi and colleagues enhances the potential for robotic interventions in thymic tumors [10] and explores innovative approaches to robotic surgery for lung cancer [11].

Subsequent studies present long-term results of robotic lobectomy for lung cancer, supporting the use of the robotic approach as a viable oncologic option [12].

In May 2009, Veronesi and colleagues reported their technique for RATS lung lobectomies [10], opening the application of robotic surgery also in thoracic surgery for major anatomical resection. They used a DaVinci Robotic System (Intuitive Surgical, Inc., Mountain View, CA, USA) with three ports plus one utility incision, used to isolate hilum elements and perform vascular and bronchial resection (using standard endoscopic staplers); subsequently, they performed standard mediastinal lymph node dissection. For dissection and isolation of the hilum structures, they used a hook and two Cadiere forceps (Intuitive); the hook was manipulated by the first robotic arm, from the utility thoracotomy access, and one of the Cadiere forceps was used to retract the lung and expose the hilum. Since this first report, other techniques have been described, including the use of different robotic systems beyond the DaVinci platform, further expanding the applicability of robotic thoracic surgery.

They can be divided into two groups: the robot-assisted techniques, which have three or four ports plus a service mini-thoracotomy, and the robot-performed techniques, with three to five ports, including a small service-access to allow the exit of the operating sample.

At the moment, only two robots are approved for the use in Thoracic surgery in Europe: The DaVinci system (the last model of the series is the DaVinci Xi, Intuitive Surgical, Inc., Mountain View, CA, USA) and the Versius Surgical Robotic System (CMR Surgical Ltd., Cambridge, UK).

The DaVinci Xi is the fourth generation of DaVinci robots. This kind of robot helps the surgeon with dedicated instruments and the possibility to train on virtual simulators. It has three parts:

– The surgeon console, where the first surgeon stays, with an advanced 3D visor.
– The vision cart, which allows the control of the robotic system.
– The patients’ cart, with four arms that can be adapted to the patient’s position.

The Versius System, introduced in 2022, is composed of a console for the surgeon that can be adapted to the surgeon for maximum comfort, an optical arm, and three arms that can be used with a lot of surgical instruments. The advantages described by the producers are that Versius can be used for integrated VATS and RATS procedures, the modular structure allows an easy allocation in the operatory room, and offers the surgeon the ability to perform the intervention closer to the operative table with respect to other robots. Because the robots’ arms are not joined to the robot, all the different settings of the operating room are feasible.

The last innovation in robotic lung surgery was reported in 2022 by Diego Gonzales Rivas [13]: he proposed an uniportal-RATS approach for lung resections, using a DaVinci Xi system (Intuitive Surgical, Inc., Mountain View, CA, USA). According to his paper, single intercostal incision, without rib spreading, using the robotic camera, robotic dissecting instruments, and robotic staplers simplifies the management of the surgical intervention. Since 2022, Rivas and colleagues have published several papers about uniportal RATS, for example, for advanced cancers, reporting 30 sleeve resections [14], for aspergilloma [15]. They also made a review [16] between single and multiportal RATS, concluding that they are similar for surgical outcomes and postoperative complications. Being an innovative technique, there are not yet papers about the long-term oncological outcomes, so we must wait for this kind of results to validate that technique. With growing experience and increased use of robotic platforms in surgical practice, many advanced instruments have been developed and released to enhance the capabilities of robotic procedures, including a wide array of port sizes and specialized robotic tools.

The bases for the instruments are the ports: there are 5-, 8-, and 12 mm ports for robotic use. The 5- and 8 mm ports allow the introduction of the instruments, the 8 mm is the camera port, while the 12 mm ports are for the introduction of staplers. There are 2 different configurations of cameras: 0° and 30°.

Several instruments are now available to allow the surgeon the best choice:

– Monopolar cautery instruments, like different-sized hooks and monopolar scissors.
– Bipolar graspers, with different-shaped and sized branches (like “Maryland” branches, fenestrated bipolar, longer or shorter graspers).
– Robotic clip appliers, with the possibility to choose between small, medium, and large clips.
– Needle drivers, for robotic sutures.
– Robotic graspers, with different branches and sizes for different uses.
– Robotic scissors.
– Other special instruments.
– Robotic staplers, with the possibility to choose vascular or parenchymal staplers.

Over the years, numerous authors have described a wide range of surgical procedures performed using RATS, including both anatomical and non-anatomical lung resections that have been extensively investigated in the literature.

Nowadays, the state-of-the-art for non-small cell lung cancer is the minimally invasive surgical approach, performing VATS or RATS, as reported in the latest guidelines. The last version of NCCN guidelines [17] says that “Minimally invasive surgery (VATS or robotic-assisted approaches) should be strongly considered for patients with no anatomic or surgical contraindications, as long as there is no compromise of standard oncologic and dissection principles of thoracic surgery” and that “Studies of robotic-assisted pulmonary resection show non-inferiority to traditional VATS approaches when performed by experienced robotic surgeons”.

With the growing experience in performing pulmonary resections using RATS, many authors have begun to extend surgical indications to include advanced lung cancers and locally invasive neoplasms. One of the milestones is the paper by Cao and colleagues (2018) [18] reporting the outcomes of mini-invasive sleeve resections, using RATS and VATS approaches. There are several papers reporting overall of more than 1000 patients, agreeing on the non-inferiority of RATS techniques. An interesting paper, by Zirafa and colleagues (2023) [19], reports 131 advanced NSCLC that showed that in the RATS group, there were fewer complications, the hospitalization time was shorter, and the overall survival was greater. The techniques are better described in the book “Pulmonary Sleeve Resections”, by Lee and Razi (2023) [20].

About chest wall resections, there is an American report [21] of 102 patients (6 domestic and 96 patients from the National Cancer database): robotic chest wall resection was feasible and was performed with acceptable short- and long-term outcomes and with a small number of complications. There are also other case reports published [22], but a particular focus should be given to a couple of reviews about the robotic resection of the first rib in the thoracic outlet syndrome [23] and the chest wall resections [24]: in these papers, the authors well explained the surgical technique.

Nowadays, one of the hot topics in thoracic surgery is the feasibility of the surgical intervention for advanced NSCLC using minimally invasive techniques. The first papers about this were published in 2021 [25]: the initial data collections reported that the early outcomes and oncological results of N2-patients after robotic lobectomy were similar to those who had open surgery. Shahin and colleagues [26] reported their experience with robotic resections for stages IIB-IVA: they reported that RATS is safe and feasible also in advanced-stage NSCLC. Kocher and colleagues [27] also reported the resection of LUL and chest wall in a Pancoast tumor, showing that even the more difficult resections can be performed by an experienced robotic surgeon. There are also a lot of papers speaking about the feasibility of minimally invasive surgery in NSCLC after neoadjuvant radio-, chemo-, and immunotherapy. All the works agree that this kind of surgery is feasible but can be more difficult, so the resection must be performed by an experienced surgeon, and the entire OR team must be ready to convert to thoracotomy. These few examples have shown that the actual line is to start the intervention with a minimally invasive technique. Some Authors have also reported that RATS is also feasible like savage surgery after neoadjuvant therapy with TKI [28], also if they suggest that it has to be performed by an experienced surgeon for the high risk of adhesions in these patients. According to some authors, the conversion rate in RATS is lower than VATS [29]: they have included more than 9500 lobectomies for N + NSCLC after chemo- and radiotherapy, finding comparable surgical and oncological outcomes between the techniques except for a lower conversion rate to thoracotomy in the RATS group.

Regarding the benign diseases, minimally invasive techniques should be encouraged. Although these conditions are not the primary focus of thoracic surgical research, it is important to recognize that, in cases of benign pathology-such as pulmonary sequestration [30] or hamartomas [31]—a minimally invasive approach is strongly recommended. Resections using such techniques have been reported in the literature since 2014.

In 2023, the Los Angeles transplantation group reported the first human robotic lung transplantation, performed using the DaVinci Xi in a 69-year-old recipient [32]. Although only a few papers have been published on this topic, there is growing interest in the potential role of minimally invasive surgery in lung transplantation [33].

## 3. Robotic Surgery in Advanced Tumors

In the last decade, the surgical treatment of lung cancer has increasingly shifted to a minimally invasive approach [34]. Minimally invasive surgery (MIS) has already proven to be superior in the management of early-stage disease in terms of long-term post-operative pain, complication risks, length of stay, and improved physical functioning. MIS has also led to a potential improvement in the 5-year overall survival, along with a comparable progression-free survival [35]. However, there is very little evidence of the use of minimally invasive techniques in the treatment of locally advanced lung cancer, where the open approach is still the most commonly used technique [36]. A locally advanced tumor is defined by a mediastinal localization (N2 disease), the size of the lesion, the invasion of structures by the primary lesion, such as vessels, chest wall, or a hilar location. Robotic surgery represents a potential alternative to open surgery in the treatment of locally advanced tumors thanks to the magnified three-dimensional vision, over than the precision of the instruments.

So far, thoracotomy has been the preferred approach for the treatment of NSCLC N2 [37,38]. Some observational studies, such as the analysis by Herb of the American national database from 2010 to 2016, have reported an increasing trend over the years in the number of cases operated with a minimally invasive technique [37]. Furthermore, according to Herb, patients who underwent MIS had a shorter recovery (1 day less compared to thoracotomy), a lower odds of 90-day and 5-year mortality as compared to open resections. Among the lobectomies for locally advanced N2 NSCLC, robotic and VATS techniques appear safer and more effective than open surgery, offering a range of both short- and long-term advantages [38]. Similar results had previously been observed in a multicenter analysis where authors concluded that robot-assisted lobectomy is safe and effective in patients with Stage III lung cancer with low conversion and complication rates, as well as a similar survival to that reported for open surgery [39].

**Figure 1:**
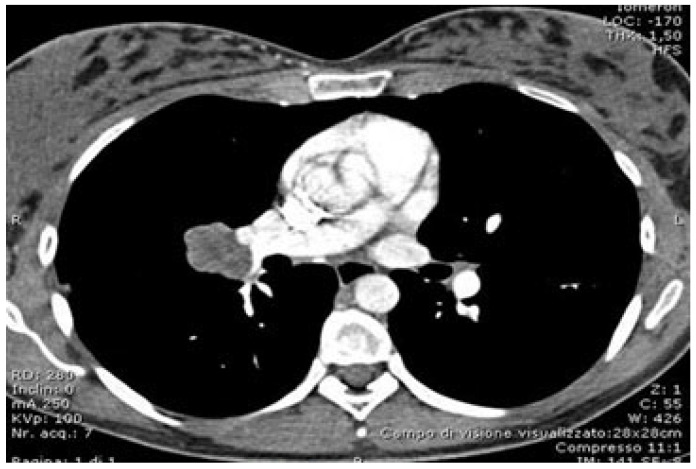
CT scan showing the target lesion.

In recent years, the use of a robotic platform has been implemented in a variety of resections: bronchoplasty, sleeve resections, pneumonectomies, and chest wall resections [40,41,42,43,44,45]. Various experiences have shown the feasibility of minimally invasive bronchoplasty and bronchial sleeve resections. Moreover, it is a fairly consolidated awareness that robotic surgery is a simpler technique compared to VATS. One of the reasons is the reduction in the VATS “fulcrum effect” owing to the presence of straight, non-articulated instruments, a better visualization, improved ergonomics, and a more precise tissue manipulation. A study published by Qiu in 2020 compared the perioperative outcomes of sleeve pulmonary lobectomy among all three techniques: robotic, open, and VATS. A higher mortality was observed among the VATS cases, while the robotic group registered a lower bleeding loss, early chest drain removal, and a shorter surgical time [42]. The advantage of robotic surgery in bronchoplasty or bronchial sleeve compared to VATS is an easier visualization of the bronchial stump and maneuverability of the needle holder with the EndoWrist instruments (Intuitive Surgical, Inc., Mountain View, CA, USA). Furthermore, the use of a self-locking wire allows for blocking the wire and keeping the suture tighter compared to the classic monofilament, especially in the case of anastomosis (Figure 1).

**Figure 2:**
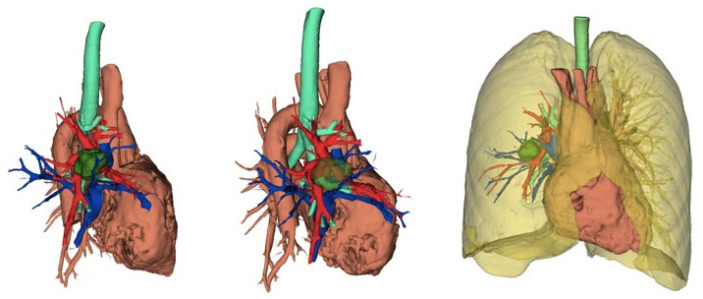
Three-dimensional reconstruction showing the anatomical relationships of the tumor with the thoracic structures.

In the literature, there is still little data concerning robotic pneumonectomies. In 2021, Patton and colleagues described a series of 13 patients who underwent robotic pneumonectomy. Of these, 8 procedures were carried out with a minimally invasive approach, whereas 5 cases were converted to thoracotomy. The average hospital stay for robotic pneumonectomies was 4 days, while for converted cases it was 7 days. The duration of surgery was significantly longer in converted cases (226 min vs. 374). All conversions occurred for anatomical reasons, not because of hemorrhage [31]. In 2021, Scheinerman described an anterior approach to robotic pneumonectomy with the isolation of the inferior pulmonary vein followed by that of the superior pulmonary vein. In left pneumonectomies, it is necessary to perform an extensive lymphadenectomy in order to isolate the main bronchus at the origin and reduce to a minimum the length of the stump [43]. In a recent paper, Louie concluded that robotic pneumonectomy requires further development, particularly in terms of management of the pulmonary artery and of the evaluation of the bronchial stump length on the left. Finally, robotic pneumonectomy should be reserved for centers with considerable experience [44] (Figure 2).

Although the available data remain limited compared to other thoracic procedures, an increasing number of reports have documented the use of robotic platforms in chest wall resections. Verm and colleagues recently described a case series of six patients affected by lung cancer infiltrating the chest wall who underwent a completely robotic resection. Their case series was compared with data from a national database that included 96 cases of robotic chest wall resections [21]. The authors demonstrated the feasibility of the technique with an acceptable level of perioperative morbidity. In particular, it is interesting to observe how the use of the robot for this type of resection has increased over time. In terms of technique, the authors suggested starting with the hilar lung resection to then moving to the thoracic wall. Regarding wall resection, they described different possibilities of rib resection. In their case series, rib shears, Gigli saw, and Kerrison rongeur were used. Overall, the best technique is chosen according to the position of the rib that must be resected. In terms of the number of ribs, authors declare that no more than two ribs were resected, mainly due to the fact that chest wall reconstruction was never performed in the reported cases. In the literature, there are no descriptions as yet of wall reconstruction performed with a robotic approach, while there are a few reports available regarding minimally invasive thoracoscopic resections [44]. In our unpublished experience, wall resection for infiltrating lung cancer was performed in a similar manner. Initially, lobectomy with an anterior approach was carried out, and the time to perform rib resection depended on the location and on the need to mobilize the lung (Video S1). This allows for the maintenance of an intact muscular-cutaneous plane and therefore reduces the surgical insult. Other approaches described in the literature are hybrid resections for Pancoast tumor in which the first rib is sectioned through an open transmanubrial approach, followed by a robotic completion lobectomy. This modality should reduce trauma and the post-operative morbidity rate [46].

To conclude, the use of the robotic approach for locally advanced tumors is already the standard technique in centers with extensive experience in robotic surgery. Training should be as comprehensive as possible, and therefore, it should be carried out in high-volume centers. Finally, the advantages of the robotic technique are related to a lower rate of post-operative complications, faster access to post-operative adjuvant therapies, and a better post-operative quality of life.

## 4. Robotic Surgery After Neoadjuvant Therapy

Robotic surgery, when incorporated into the management of locally advanced non-small cell lung cancer (NSCLC) following induction treatments, represents a significant technological advancement in minimally invasive surgical approaches, providing clear advantages over traditional methods. This surgical modality, characterized by enhanced visualization (3D versus 2D) and precise instrument movement exceeding human capabilities, facilitates shorter and more intricate procedures, including radical lymphadenectomy, bronchoplasty, and vascular dissection. Beyond the operating room, patients benefit from accelerated postoperative recovery, enabling the commencement of full-dose adjuvant chemotherapy within a prudent timeframe, typically within six weeks after surgery.

Studies, including a retrospective analysis by Park and colleagues involving 428 locally advanced NSCLC patients who underwent induction chemotherapy followed by lobectomy, highlight comparable oncological outcomes between open thoracotomy and minimally invasive techniques, with a significantly shorter hospital stay observed in the latter group [47]. Veronesi and collaborators contribute an international perspective, reporting on 223 patients undergoing robot-assisted resection for stage IIIA–N2 NSCLC, of which 34 underwent neoadjuvant treatment. Among those receiving neoadjuvant chemotherapy, all achieved R0 resection, and although 15% required conversion, none did so due to bleeding, with 12% experiencing Grade III or IV postoperative complications. The study underscores the efficacy of robotic surgery in achieving a high rate of radical resection (98.4%) within this patient subset [39].

Despite its advantages, robotic surgery does have limitations, including contraindications for specific procedures such as intrapericardial pneumonectomy, atrial resections, major vascular resection, and reconstruction. Challenges akin to open surgery are posed by large central tumors, multi-station lymph node compromise, and prior irradiation.

In a detailed analysis of surgical outcomes, Zirafa et al. discuss their experience with robotic lung resections in locally advanced NSCLC, where 12.5% of patients received neoadjuvant treatment. The authors report an 18% rate of postoperative complications, mainly prolonged air leaks and postoperative bleeding/anemia. Interestingly, they note variations in outcomes with different generations of robotic platforms, underscoring the importance of evolving technology in refining surgical approaches [19].

Casiraghi et al., in a prospective study analyzing robotic surgery after neoadjuvant treatment, highlight compelling outcomes associated with robotic lobectomy compared to open surgery. The robotic group exhibited a significantly shorter median resection time, with no significant differences in lymph node resection and positivity. Moreover, there were no notable distinctions in progression-free survival (PFS) or overall survival (OS). These findings collectively suggest that early outcomes and oncological results for N2-patients undergoing robotic lobectomy were comparable to those treated with open surgery [25].

In conclusion, the evolving landscape of robotic surgery in locally advanced NSCLC post-induction treatments provides compelling evidence of its efficacy and benefits. While acknowledging technical advancements and improved patient outcomes, careful consideration of patient selection and procedure-specific contraindications remains imperative for the successful integration of robotic surgery into the comprehensive management of locally advanced NSCLC.

## 5. Preoperative Evaluation and Software in Thoracic Surgery

In recent decades, the indication for minimally invasive surgery has increased tremendously in the thoracic field, even in highly complex cases. In parallel, technological evolution has allowed the surgeon to be more precise and to personalize the procedures. For instance, three-dimensional CT reconstructions have been widely used since the 2000s for different purposes.

In 2013, the study by Ikeda and colleagues showed the importance of several computerized software programs for virtual three-dimensional reconstruction of chest CT scans in the case of lung cancer [48]. The study documented the usefulness of this technology to precisely localize the lung lesion, to study the vessels and the tracheobronchial tree, and also to plan the positioning of the trocars in case of VATS (video-assisted thoracic surgery).

In 2016, Toyofumi has implemented the use of three-dimensional reconstructions starting from multi-detector computed tomography, as well as in sublobar resections, also in the planning of EBUS (endobronchial ultrasonography) -TBNA (transbronchial needle aspiration) and in 3D-CT-angiography in the case of living donor lobar lung transplantations, in order to plan surgery with extreme precision [49].

### 5.1. Preoperative Evaluation and Software in Robotic Surgery

One of the most critical components for success in robotic-assisted thoracic surgery (RATS) is represented by preoperative evaluation, augmented by advanced planning software. These preparatory steps aim to optimize surgical outcomes by improving surgical planning, patient selection, and procedural accuracy. The preoperative evaluation has paramount importance in thoracic robotic surgery due to the complexity of the anatomy and the need for precise knowledge of vital structures such as the lungs, major blood vessels, and nerves. The pre-operative anatomic study involves comprehensive imaging studies. High-resolution computed tomography (CT) or magnetic resonance imaging (MRI) allows surgeons to assess tumor location, size, and relationship to adjacent anatomical structures. Additionally, they give information about the possible involvement of lymph nodes, which is essential for staging in lung cancer and other thoracic malignancies [50].

Pulmonary function tests are an integral part of the preoperative workup, particularly for lung resection candidates. Such evaluations determine the patient’s ability to tolerate partial lung removal and are predictive of postoperative recovery potential [51]. Furthermore, cardiopulmonary assessments, including echocardiograms and stress tests, are often necessary to assess cardiac reserve, especially in elderly patients or those with significant comorbidities [52]. The combination of these assessments allows surgeons to select optimal candidates for robotic procedures, minimizing risks and maximizing outcomes.

### 5.2. Role of Advanced Software in Preoperative Planning

Modern planning software has introduced an unprecedented level of accuracy in robotic thoracic surgery. Three-dimensional (3D) reconstruction and virtual simulation software, such as Synapse 3D (version 3.0) and Materialize Mimics (Materialize), facilitate an in-depth understanding of each patient’s unique anatomy. These tools create a 3D model based on preoperative CT or MRI images, which enables surgeons to virtually explore the operative site, plan incisions, and determine the most appropriate pathway for the robotic instruments. This technology is particularly valuable in complex cases involving lung cancer, where a precise understanding of the relationship between the tumor and surrounding vasculature can significantly impact surgical decision-making.

Some software systems integrate augmented reality (AR) features, overlaying 3D models onto live patient images during surgery. This approach helps the surgeon visually correlate preoperative imaging with real-time anatomy, which is useful in navigating challenging areas or confirming anatomical landmarks during bronchoscopy [53]. By using AR, robotic thoracic surgeons can work with greater confidence and precision [54], minimizing intraoperative risks associated with poor visualization of critical structures.

Despite its promising applications, AR in thoracic surgery currently faces several limitations. These include the high cost of equipment, limited integration into standard operating room workflows, and technical challenges in achieving real-time image alignment with sufficient precision. Moreover, the adoption of AR requires dedicated training and familiarity with specialized software, which may pose a barrier for widespread use. The accuracy of AR overlays can also be compromised by intraoperative anatomical shifts or changes in patient positioning. As such, while AR has significant potential to enhance surgical precision, its routine use in robotic thoracic procedures remains constrained by these practical and technological hurdles, at the moment.

### 5.3. Navigation and Enhanced Visualization

Robotic systems like the da Vinci Surgical System allow for high-definition, magnified visualization, yet integrating preoperative imaging into the surgical console improves the surgeon’s understanding of spatial relationships in real-time. This integration is further enhanced by software-driven navigation systems such as LungPoint and superDimension. These technologies allow for the mapping and tracking of surgical instruments within the 3D model of the patient’s anatomy, providing real-time guidance and minimizing guesswork during complex resections [55]. Enhanced navigation tools are particularly valuable in robotic thoracic surgery, where precision is essential to avoid complications around sensitive thoracic structures.

### 5.4. Predictive Analytics and Artificial Intelligence

Predictive analytics and artificial intelligence (AI) are increasingly incorporated into preoperative software to help estimate potential outcomes and assess surgical risks. Some platforms use algorithms that analyze patient data, including demographic information, comorbidities, imaging findings, and previous surgical history, to predict postoperative outcomes and potential complications. These predictive models aid surgeons in risk assessment, enabling better patient counseling and shared decision-making. For instance, AI can predict potential respiratory complications or prolonged hospital stays, which helps both the surgical team and patients in preparing for recovery and postoperative management [56]. With profound effects on patient care, artificial intelligence (AI) in radiomics has become a transformative force in contemporary medicine, particularly within robotics in thoracic surgery. Radiomics, which involves quantitative feature extraction and analysis from medical images, offers valuable imaging biomarkers that provide insights into disease characteristics, treatment responses, and patient outcomes. The integration of AI techniques in radiomics—such as machine learning and deep learning—has enabled the development of advanced computer-aided diagnostic systems, predictive models, and decision support tools. In thoracic surgery, the synergy between AI-enhanced radiomics and robotic systems is enhancing surgical precision, preoperative planning, and intraoperative decision-making. By analyzing imaging data in real time, AI-driven radiomics can guide robotic-assisted procedures, helping to accurately localize tumors, assess their heterogeneity, and predict patient-specific responses to interventions. This combination of robotics and AI not only boosts diagnostic and therapeutic accuracy but also minimizes invasiveness, leading to improved outcomes, reduced recovery times, and more personalized patient care in thoracic surgery [57,58].

### 5.5. The Impact of Software on Workflow and Training

Preoperative software also plays an important role in enhancing surgical workflow and training. Simulating the steps of a robotic thoracic procedure allows surgeons to practice the procedure virtually, refining their approach before entering the operating room. For instance, the use of robotic simulators enables novice surgeons to familiarize themselves with the console and robotic arms, leading to a smoother and more efficient operative experience [59]. Experienced surgeons can also benefit by rehearsing complex cases, reducing their operative times, and enhancing precision.

In addition, virtual platforms for team training and communication improve intraoperative coordination. For instance, digital platforms enable team members to review planned surgical steps and familiarize themselves with the unique aspects of a case. This preparatory work facilitates smoother interactions among the surgical, anesthetic, and nursing teams, ultimately reducing the potential for errors and enhancing overall surgical efficiency [50].

## 6. Conclusions

Robotics represents one of the most transformative innovations in the field of oncological surgery. Its immense potential for enhancing surgical efficacy is undeniable, and many of its current limitations—such as high costs, extended operative times, and possibly the lack of tactile feedback—are expected to be addressed in the near future.

Advancements in robotic surgery for locally advanced non-small cell lung cancer (NSCLC), including cases following neoadjuvant treatment, have shown significant benefits. These include improved surgical precision, reduced recovery times, and oncological outcomes comparable to those achieved with traditional methods.

The integration of advanced preoperative evaluation and planning tools, such as 3D imaging and AI-driven analytics, further enhances surgical techniques by optimizing patient selection and increasing procedural accuracy. These technologies enable superior visualization, precise navigation, and real-time decision-making during complex surgeries, ultimately leading to better patient outcomes.

As robotic systems and preoperative software continue to evolve, they pave the way for more personalized and effective treatment strategies in thoracic surgery. This progress underscores the critical importance of meticulous patient selection and procedure-specific planning, ensuring that these cutting-edge technologies deliver maximum benefit.

## Supplementary Materials

The following supporting information can be downloaded at: https://www.mdpi.com/article/10.3390/curroncol32050244/s1, Video S1. Robotic Right Upper Lobectomy and Chest Wall Resection.
